# Functional tunability from a distance: Rheostat positions influence allosteric coupling between two distant binding sites

**DOI:** 10.1038/s41598-019-53464-z

**Published:** 2019-11-18

**Authors:** Tiffany Wu, Liskin Swint-Kruse, Aron W. Fenton

**Affiliations:** Department of Biochemistry and Molecular Biology, The University of Kansas Medical Center, Kansas City, KS 66160 USA

**Keywords:** Kinases, Personalized medicine

## Abstract

For protein mutagenesis, a common expectation is that important positions will behave like on/off “toggle” switches (*i.e*., a few substitutions act like wildtype, most abolish function). However, there exists another class of important positions that manifests a wide range of functional outcomes upon substitution: “rheostat” positions. Previously, we evaluated rheostat positions located near the allosteric binding sites for inhibitor alanine (Ala) and activator fructose-1,6-bisphosphate (Fru-1,6-BP) in human liver pyruvate kinase. When substituted with multiple amino acids, many positions demonstrated moderate rheostatic effects on allosteric coupling between effector binding and phosphoenolpyruvate (PEP) binding in the active site. Nonetheless, the combined outcomes of all positions sampled the full range of possible allosteric coupling (full tunability). However, that study only evaluated allosteric tunability of “local” positions, *i.e*., positions were located near the binding sites of the allosteric ligand being assessed. Here, we evaluated tunability of allosteric coupling when mutated sites were distant from the allosterically-coupled binding sites. Positions near the Ala binding site had rheostatic outcomes on allosteric coupling between Fru-1,6-BP and PEP binding. In contrast, positions in the Fru-1,6-BP site exhibited modest effects on coupling between Ala and PEP binding. Analyzed in aggregate, both PEP/Ala and PEP/Fru-1,6-BP coupling were again fully tunable by amino acid substitutions at this limited set of distant positions. Furthermore, some positions exhibited rheostatic control over multiple parameters and others exhibited rheostatic effects on one parameter and toggle control over a second. These findings highlight challenges in efforts to both predict/interpret mutational outcomes and engineer functions into proteins.

## Introduction

**Figure 1:**
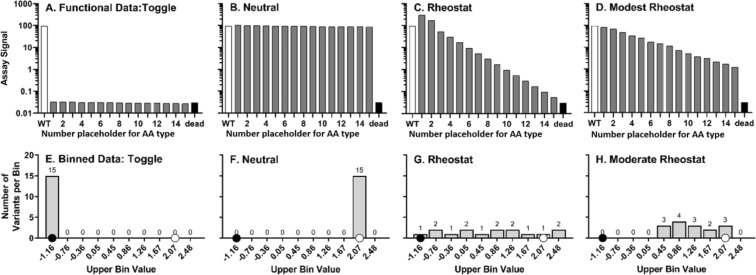
Simulated examples of neutral, toggle, rheostat, and modest rheostat positions. Panel (A) shows a toggle position for which all substitutions abolish function: this simulated example gives rise to the maximum possible toggle score. Panel (B) shows a neutral position, for which most substitutions are like wildtype. Panel (C) shows a rheostat position for which substitution values range from wildtype to “dead”. An example that does not perfectly conform to the rheostat profile but nevertheless exhibits rheostat-like properties^3^ is shown in panel (D). For subsequent histogram analyses, this range is first divided into bins; here, the histograms in panels (E–H) correspond to the simulated data in panels (A–D). On each histogram, a white dot is used to indicate the bin that includes wildtype data and a black dot is used to indicate the bin that corresponds to “dead” (*e.g*., “no allostery”) function. The values on the x-axis indicate the upper-bound value of a bin in which all variants possess logarithmic transformations of functional value lesser than or equal to the label of the bin and neither lesser nor equal to the next smaller bin.

In mutagenesis studies that evaluate the contributions of important amino acid positions to protein function, our collective experience has led to a common expectation: For a given position, a few substitutions will result in wildtype-like function, but most substitutions will abolish function (*i.e*., function is “toggled” on or off like a light switch). However, Gray *et al*.^1^ showed that most studies on which our expectations are based have focused on conserved positions, which biases our collective experience. Recently, we selected a broader range of positions for mutagenesis and in doing so, we identified another type of mutagenesis outcome: When these positions were substituted with various amino acids, a wide range of functional outcomes was obtained. When rank-ordered, the outcomes showed a continuum of functional magnitudes, analogous to electronic rheostats (*e.g*., a dimmer switch)^2^. We further found that a histogram analyses can be used to quantify the degree of rheostat character^3^ (Fig. 1).

Once rheostat positions were recognized to exist, we initiated studies to evaluate the prevalence of rheostat positions across a range of proteins with different, quantifiable functions^3^. One such protein was human liver pyruvate kinase (hLPYK), a model system for allosteric regulation. The affinity of hLPYK for its substrate in the active site is regulated by two different allosteric effectors that bind at two allosteric sites that are both distinct from the active site. Thus, rheostat character was evaluated using five functional parameters associated with hLPYK functions^4–6^:

$$\begin{array}{c}\begin{array}{ll}{K}_{a-PEP} & {\rm{PEP}}\,{\rm{apparent}}\,{\rm{affinity}}\\ {K}_{ix-Ala} & {\rm{Ala}}\,{\rm{binding}}\\ {K}_{ix-FBP} & {\rm{Fru}} \mbox{-} 1,6 \mbox{-} {\rm{BP}}\,{\rm{binding}}\\ {Q}_{ax-Ala} & {\rm{Allosteric}}\,{\rm{coupling}}\,{\rm{between}}\,{\rm{PEP}}\,{\rm{binding}}\,{\rm{and}}\,{\rm{Ala}}\,{\rm{binding}}\\ {Q}_{ax-FBP} & {\rm{Allosteric}}\,{\rm{coupling}}\,{\rm{between}}\,{\rm{PEP}}\,{\rm{and}}\,{\rm{Fru}} \mbox{-} 1,6 \mbox{-} {\rm{BP}}\,{\rm{binding}}{\rm{.}}\end{array}\end{array}$$

In our first study of hLPYK rheostat positions, we evaluated positions with side-chains that directly interacted with or were very close to the bound allosteric effector^7,8^. Those previous studies included monitoring local effects; that is, we evaluated substitution effects on binding and allosteric regulation of the allosteric ligand with which the positions directly interacted (*e.g*., positions in the Fru-1,6-BP binding site were evaluated for effects on PEP/Fru-1,6-BP allosteric coupling). When scored for their rheostat character^3^, most of the individual positions in hLPYK allosteric sites had modest rheostat scores for either binding of their allosteric ligand or for allosteric coupling between that effector and substrate binding.

**Figure 2:**
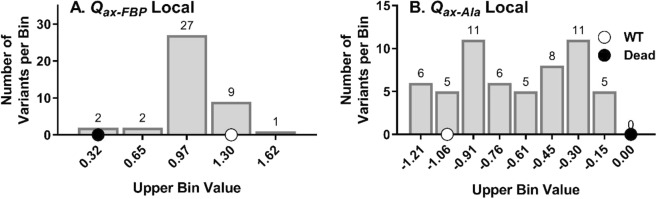
Binning of “local” allosteric coupling outcomes. These functional data were taken from a previous study^3^ and compile the allosteric coupling values  obtained for all substitutions at positions in each of the two allosteric binding sites. This histogram presentation is used here to demonstrate the number of histogram bins [spanning the region between no allostery/“dead” (black dot) and wildtype allosteric coupling (white dot)] that are occupied with at least one entry (see numbers above each bin). The high level of occupancy demonstrated in this local-position data set is defined herein as “tunability.” All substitutions at seven positions in the Fru-1,6-BP binding site were evaluated for their effects on PEP/Fru-1,6-BP allosteric coupling (*Q*_*ax-FBP*_), and all substitutions at eight positions in the Ala binding site were evaluated for their effects on PEP/Ala allosteric coupling (*Q*_*ax-Ala*_). Data shown here are a compilation of the measurements of allosteric coupling obtained for all substitutions at all positions in each of the allosteric binding sites.

Beyond the evaluation of single positions, when outcomes for all mutated positions from one allosteric site were combined into a single histogram analysis, high rheostat scores were calculated for both allosteric coupling parameters (Fig. 2). This led us to refine our definitions: A “rheostat position” is a single position for which substitutions achieve a full range of functional outcomes between the absence of function (“dead”) and “better than wildtype”. “Full tunability” describes the scenario in which a complete range of functional outcomes can be obtained by substituting a subset of positions. In histogram analyses, tunability increases as more bins are populated by at least one entry (Fig. 2). Note that full tunability (all bins having at least one entry) can be obtained even if the individual component positions are modestly or poorly rheostatic.

Our previous study identified full tunability^7,8^. Due to the design of that previous study, a conclushion can be made that full tunability in allosteric function can be achievable by substituting positions in the respective allosteric binding site. Subsequently, we were curious whether allosteric coupling could be fully tunable when substituted positions were distant from the two allosterically-coupled binding sites. In hLPYK, this could be assessed by considering whether substitutions in the Fru-1,6-BP binding site altered coupling between PEP and Ala, and *vice versa*. Here, we show tunability was again high from a small set of positions even though those positions were distant from the two allosteric sites being studied. Furthermore, several positions exhibited rheostat control over multiple parameters. Finally, two positions simultaneously acted as a rheostat for one parameter and a toggle for a second parameter. This range of outcomes highlights challenges that can arise when predicting/interpreting mutational outcomes and rationally engineering functional variation into existing proteins. In particular, efforts to engineer allosteric control^5,6,9^ over a range of functions^10–14^ will benefit from improved understanding of how distant residues can control allosteric coupling between two binding events.

## Materials and Methods

All methods were performed in accordance with relevant guidelines. In particular, all use of recombinant DNA was according to the NIH Guidelines and was approved by the KUMC Institutional Biosafety Committee.

**Figure 3:**
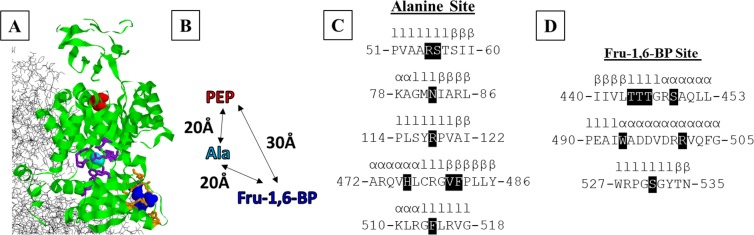
Structural features of positions tested in this study. (**A**) Locations of positions evaluated in this study. One subunit in the homotetrameric structure (PDB:4IMA^30^) is in green; neighboring subunits are in black. In this structure, citrate (red) was in the PEP binding site. An ethylene glycol molecule (cyan) resides in the Ala binding site (as defined in PDB:2G50^23^). Fru-1,6-BP was present in the structure and is colored blue. Positions evaluated for rheostatic behavior in this study are in purple (Ala binding site) and orange (Fru-1,6-BP binding site). (**B**) Distances between ligand binding sites. (**C**) Sequence and secondary structures surrounding residues in the Ala binding site. (**D**) Sequence and secondary structures surrounding residues in the Fru-1,6-BP binding site. In C and D, secondary structures are indicated as l-loop, α-helix, or β-sheet. Positions substituted in this study are shown as white text on a black background.

All hLPYK mutations were previously created^7,8^. This group of mutations include substitutions at seven positions in the Fru-1,6-BP binding site and eight positions in the Ala binding site (Fig. 3). (The distance between binding sites and the secondary structure surrounding substituted positions are also included in Fig. 3.) At each position, 9–16 substitutions were created for a total of 197 variants of hLPYK (See Supplemental Tables 2 and 3 for a list of individual substituted proteins included in this study). Protein preparation, assays of enzymatic activity, and data analysis were carried out as previously reported. In short, using substrate titrations of enzymatic activity, the apparent affinity of hLPYK for its substrate, PEP, was evaluated over a concentration range for either Fru-1,6-BP or Ala. For each allosteric ligand, the coupling constant *Q*_*ax*_ was calculated to characterize allosteric function^4,15–17^:

1 $${Q}_{ax}={K}_{a}/{K}_{a/x}={K}_{ix}/{K}_{ix/a},$$

where *K*_*a*_ is the apparent affinity for ligand A (*e.g*., substrate *K*_*a-PEP*_) in the absence of ligand X (*e.g*., effector Ala or Fru-1,6-BP); *K*_*a/x*_ is the apparent affinity for ligand A in the presence of saturating ligand X; *K*_*ix*_ is the affinity for ligand X (*e.g*., *K*_*ix-Ala*_ and *K*_*ix-FBP*_) in the absence of ligand A; and *K*_*ix/a*_ is the affinity of the protein for ligand X in the presence of saturating ligand A.

When a variant failed to demonstrate detectable activity, protein preparation was replicated at least three times before designating the protein as catalytically non-active. No additional steps were taken to concentrate protein or assay immediately after cell lysis, both of which are approaches that can better detect enzymatic activity in a destabilized mutant protein. All data were collected using activity measurements. Therefore, when variants lacked catalytic activity, other parameters were not assessed. The absence of activity itself was not evaluated because there are multiple reasons why activity might have been altered. (1) The modified enzyme could lack the ability to fold correctly. (2) The folded form of the protein may be sufficiently unstable that the protein unfolds by the time of assaying for enzymatic activity. (3) The enzyme could have a greatly reduced *K*_*m*_ for PEP, such that activity is not observed within the solubility limits of PEP. (4) The k_cat_ of the enzyme could be reduced below detectable limits.

Once affinity, binding, and allosteric coupling parameters were determined for variants at each position, the degree of rheostat character was evaluated using the RheoScale calculator^3^. For each position, this calculator uses all available variants to quantify its rheostat, toggle, and neutral character *via* histogram analyses. Rheostat scores describe how well the functional data sample the histogram bins. Toggle scores reflect the number of variants that fall into the same bin as “dead” protein. For prior experimental data^18^, toggle positions were defined when at least 64% of substitutions abolished function (*i.e*., “dead”) and at most 16% of the substitutions exhibited wildtype function. Neutral scores reflect the number of variants that fall into a histogram bin centered on the value of the wildtype protein; these calculations are extensively discussed elsewhere^19^.

To identify the appropriate width and number of histogram bins, the RheoScale calculator assessed the full experimental dataset for (i)minimal and maximal values for the parameter being assessed, (ii) average error of experimental measurements, and (iii) average number of variants available per position. Since completing our first study of “local” hLPYK rheostat positions^3^, we have obtained and collated a much larger dataset of parameters for hLPYK variants and used them to refine RheoScale histogram analyses^19,20^. These include measurements for additional variants and multiple, independent measurements of wildtype hLPYK (Supplemental Table 1). The latter was used to estimate experimental error for all variants in this study. (Prior RheoScale analyses used errors of the fit which are smaller than experimental errors.) Revised “local” scores are included in the supplement and used for comparison to the current “distant” scores. Note that neutral scores were calculated using the revised methods of^19^ for both local and distant scores.

For most of the RheoScale calculations, “local” rheostat scores showed little change. However, for *Q*_*ax-FBP*_, two factors became apparent. First, replicates of wildtype hLPYK showed that this parameter had larger experimental error than previously estimated (and larger than other parameters). This decreased the number of bins used in histogram analyses to 5 when the histogram range was delimited by wildtype and “dead” (zero allosteric coupling). Second, several additional variants had “better” *Q*_*ax-FBP*_ values than wildtype hLPYK; this expanded the maximum range of the histogram. Some of these variants had such large effects on *Q*_*ax-FBP*_, and thus on the maximal range, that if delimited by maximum score and zero, all other variants would be compressed into a small percent of bins. Empirically, we found that this under-represented the rheostat character of positions for which substitutions accessed the full range between wildtype and “dead” allosteric coupling. Thus, we chose to classify all “better than wildtype” values into a single bin and reduce the number of bins between wildtype and “dead” by 1 from the RheoScale-recommended bin number. In doing so, the number of bins used in histogram analysis remains the same as the recommended bin number. Furthermore, the bin containing wildtype was centered on the average of all wildtype measurements. This methodology of using [n-1.5] bins (where n is the recommended number of bins) for the range spanning the wildtype average to the “dead” value plus one “better than wildtype” bin was used for all parameters.

## Results

In this work, we evaluated outcomes that resulted from substituting seven positions in/near the Fru-1,6-BP site^8^ and eight positions in/near the Ala binding site^7^ (Fig. 3). At each of these positions, nine to 16 substitutions were introduced for a total of 197 substitutions. We previously reported *K*_*a-PEP*_, *K*_*ix-Ala*_, and *Q*_*ax-Ala*_ for variants in the Ala binding site and *K*_*a-PEP*_, *K*_*ix-FBP*_, and *Q*_*ax-FBP*_ for variants in the Fru-1,6-BP binding site^3^. For this work, we performed an additional 18,000 assays to determine *K*_*ix-FBP*_ and *Q*_*ax-FBP*_ for variants in the Ala binding site and *K*_*ix-Ala*_ and *Q*_*ax-Ala*_ for variants in the Fru-1,6-BP binding site (Supplemental Tables 2 and 3; Supplemental Figs. 1–4). As previously noted^3^, most substitutions at position 483 resulted in a catalytically inactive protein. Since this prevented further evaluation of the binding of effectors and allosteric coupling, this position was not considered further.

To quantify the composite functional results for the 16 positions of this study, we used the RheoScale calculator to generate three scores: a rheostat score, a toggle score, and a neutral score. All three scores range from 0 to 1, with 1 being the perfect manifestation of the substitution character (*e.g*., a perfect rheostat position). Empirically, for datasets the size of this hLPYK set, we have found that rheostat scores above 0.5 indicate strong rheostat character of the position^3^. As discussed extensively in another study, neutral scores above 0.7 are considered to be a high score^19^. For toggle scores, we previously defined a strong toggle threshold of “at least 8 severe variants”^18^ in example positions with 12–13 substitutions, effectively resulting in a RheoScale toggle score definition of 0.64 or greater. As detailed in Methods, these and other new hLPYK data were used to refine RheoScale parameters; thus previously-published data for “local” substitutions were reanalyzed with new parameters to maintain consistency in comparisons. The revised scores are reported in the supplement (Supplemental Fig. 5), as are all experimental measurements for the “distant” substitutions and histograms for individual positions (Supplemental Figs. 1–4).

**Figure 4:**
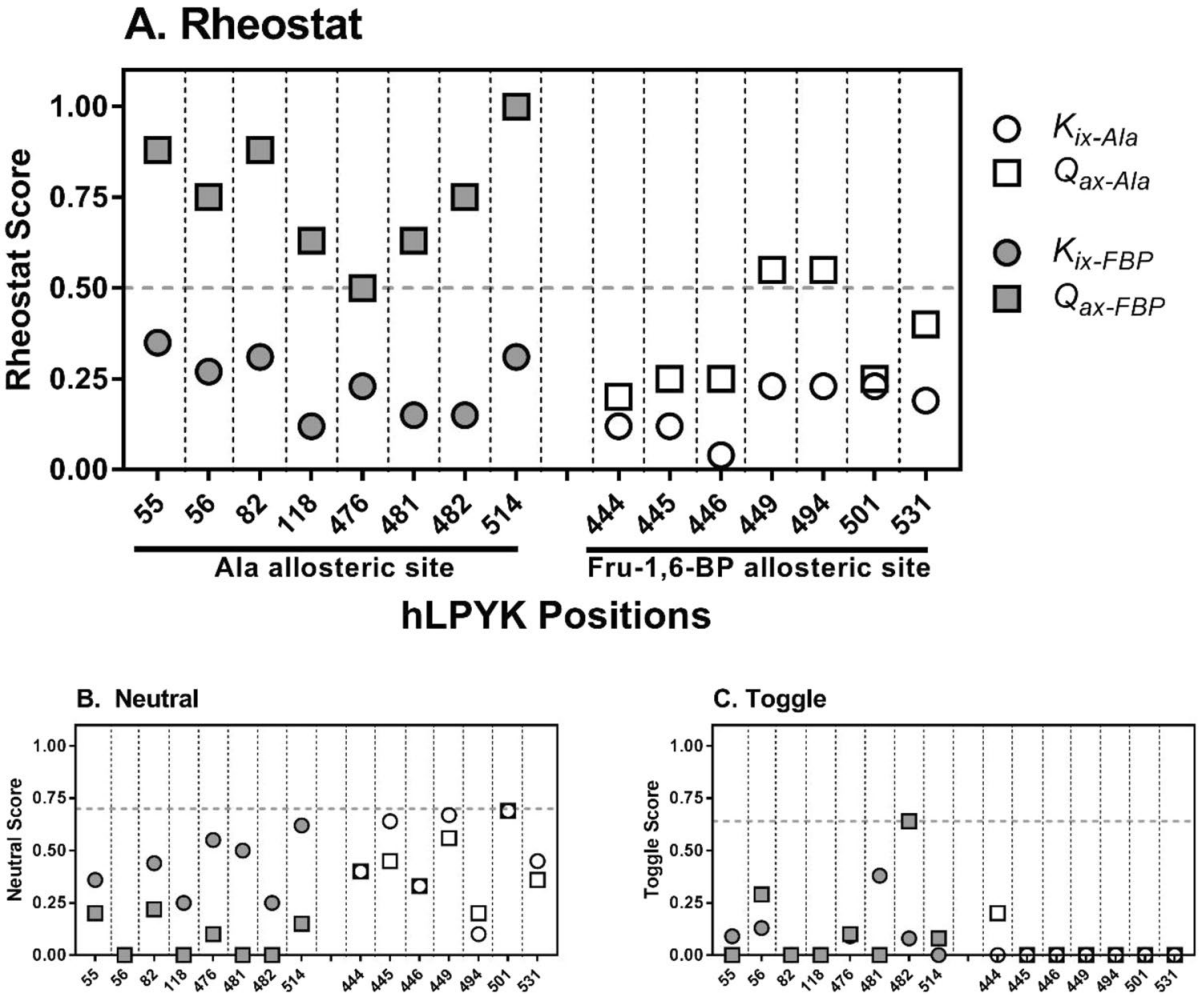
Calculated scores for substituted “distant” positions in and near the allosteric sites of hLPYK. The effects of distant substitutions in the Fru-1,6-BP binding site on Ala binding (*K*_*ix-Ala*_) and PEP/Ala allosteric coupling (*Q*_*ax-Ala*_) and the effects of distant substitutions in the Ala binding site on Fru-1,6-BP binding (*K*_*ix-FBP*_) and PEP/Fru-1,6-BP allosteric coupling (*Q*_*ax-FBP*_) were used to calculate rheostat (top), neutral (bottom left) and toggle (bottom right) scores for the hLPYK positions noted on the x-axis. The scores for the same variants on their respective “local” parameters were previously reported^3^. For direct comparison with the distant data and as described in Methods, the revised scores calculated for the local dataset are included in an all-inclusive figure in Supplemental Fig. 6 and Rheoscale scores are reported in Supplemental Table 4 for all positions and all parameters. Dashed vertical lines separate the scores for individual positions. For the rheostat scores, the horizontal dashed line at 0.5 indicates the empirically determined threshold that delineates rheostat positions. For the neutral scores, the horizontal dashed line at 0.7 to delineate high neutral scores is based on empirical comparisons in a previous study^19^. For the toggle scores, the horizontal dashed line at 0.64 corresponds to the threshold defined in^18^ to delineate a toggle position.

Analyses of the distant experimental measurements are shown in Fig. 4. On the left-hand side of each panel, the influence of positions in the Ala binding site on Fru-1,6-BP binding and PEP/Fru-1,6-BP allosteric coupling is shown. Overall, rheostat scores for *Q*_*ax-FBP*_ (most above 0.5) were notably larger than *K*_*ix-FBP*_. None of the parameters showed neutral scores above the 0.7 neutral score threshold. All toggle scores for mutants in the Ala binding site were below the 0.64 toggle score cutoff.

The right-hand side of Fig. 4 shows the influence of positions in the Fru-1,6-BP binding site on Ala binding and PEP/Ala allosteric coupling. For most positions, the rheostat scores were low for both parameters. Nonetheless, positions 449 and 494 had a rheostat score for *Q*_*ax-Ala*_ above 0.5, indicating distant control over PEP/Ala coupling. Again, the influence on allosteric coupling was often greater than effects on effector binding and none of the parameters demonstrated neutral scores above 0.7. Likewise, there was very little toggle character for positions in the Fru-1,6-BP binding site for Ala binding or PEP/Ala allosteric coupling with no scores above the 0.64 cutoff.

In summary, substitutions at two positions in the Fru-1,6-BP binding site significantly altered PEP/Ala coupling and all eight positions in the Ala binding site exhibited rheostatic control over PEP/Fru-1,6-BP allosteric coupling. Strikingly, for *Q*_*ax-FBP*_, position 514 exhibited a nearly perfect rheostat score and positions 55 and 82 had high scores. Indeed, these scores were higher than any obtained for the “local” substitution study.

**Figure 5:**
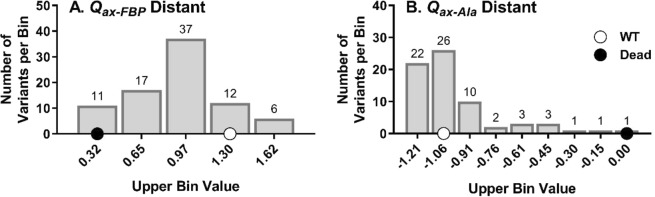
Tunability of allosteric coupling functions *via* substitutions at positions in “distant” sites. Data shown here are a compilation of the allosteric coupling values measured for all substitutions for all positions in Fig. 4. Like Fig. 2A, at least one substitution in each binding site results in one of the possible functional outcome subranges (histogram bins) that span the range between no allostery/“dead” (black dot) and wildtype allosteric coupling (white dot).

Next, we considered the overall tunability of the two allosteric coupling parameters, *Q*_*ax-Ala*_ and *Q*_*ax-FBP*_. Tunability was determined by combining data for all positions in the Ala site or all positions in the Fru-1,6-BP site (except position 483, as indicated above) for RheoScale analyses. Figure 5 shows full tunability was observed for both parameters: the “better than wildtype” bin and each histogram bin between the maximum (wildtype) and minimum (no allostery) values were occupied by at least one variant. Interestingly, similar results were obtained when the individual position with perfect rheostat control over *Q*_*ax-FBP*_ was excluded from analyses. Thus, the overall tunability of *Q*_*ax-Ala*_ and *Q*_*ax-FBP*_ was very high, even though only a small subset of the total hLPYK positions were used for analyses.

**Figure 6:**
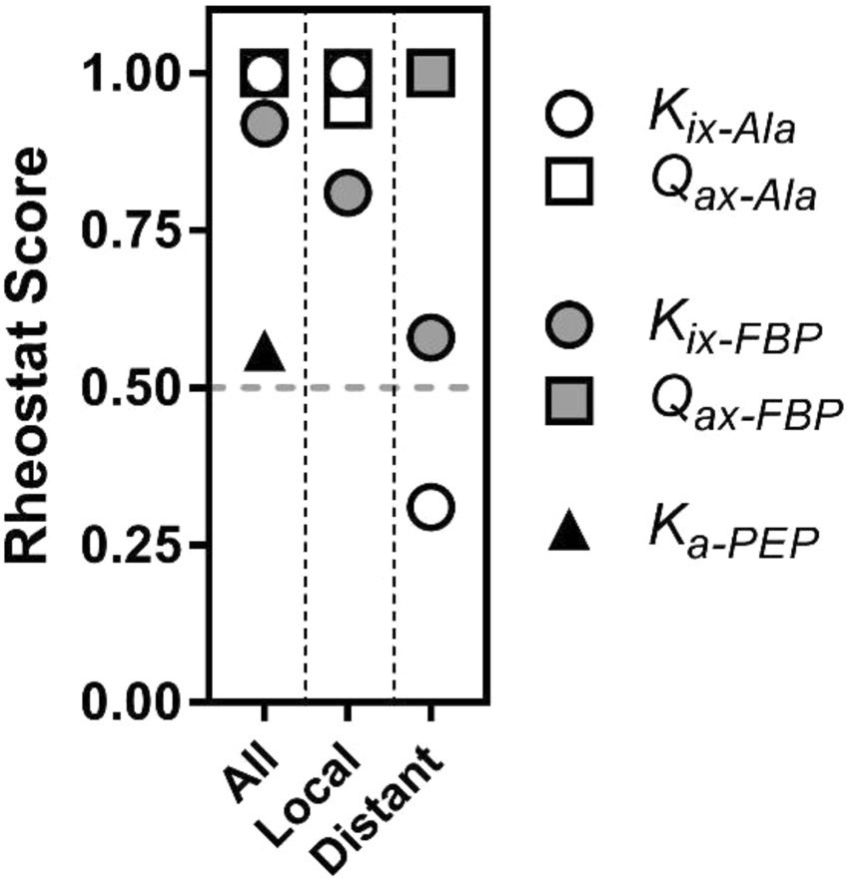
Rheostat scores for compilations of substitutional outcomes from multiple positions. When compilations of data are used to calculate a rheostat score, full tunability is reflected by a rheostat score that approaches 1. Results from both the current work and the previous study^3^ are summarized here: Compilation rheostat scores were calculated for the “local” dataset, the “distant” dataset, and the combination of both. When the symbol for *Q*_*ax-Ala*_ is not obvious in the “All” and “Distant” columns, it is behind the symbol for *Q*_*ax-FBP*_. Scores for these parameters are reported in Supplemental Table 4.

Figure 6 shows the rheostat scores calculated for the full dataset, the local subset, and the distant subset. Surprisingly, for *Q*_*ax-FBP*_, the variants located in the Ala binding site (distant) can better tune this parameter (*i.e*., scores closer to 1) than the set of variants located in the Fru-1,6-BP binding site (local). Furthermore, substitutions in the Fru-1,6-BP site (distant) can tune *Q*_*ax-Ala*_ as comprehensively as those in the Ala binding site (local).

**Figure 7:**
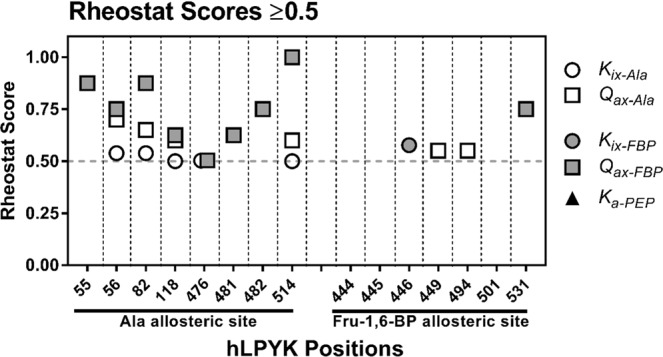
Rheostat scores greater than or equal to 0.5 for both distant and local parameters. Note that no rheostat scores for *K*_*a-PEP*_ met this threshold. Scores for these parameters are reported in Supplemental Table 4.

**Figure 8:**
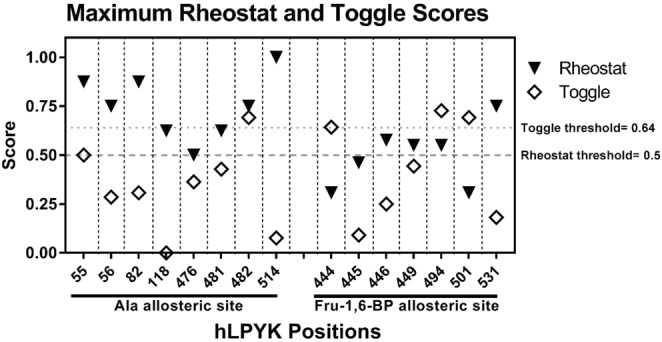
The maximum rheostat and maximum toggle scores for each position when collectively considering all 5 parameters as defined in the text. Scores for these parameters are reported in Supplemental Table 4.

We next considered additional insights these data might provide about the functional roles of the individual substituted positions. Notably, when both local and distant scores were considered, positions 56, 82, 118, 476, and 514 exerted rheostatic control over multiple parameters (Fig. 7). Due to the mathematical relationship of parameters (Eq. 1), we queried whether correlation existed between rheostatically-controlled parameters with rheostat scores above 0.5 (*e.g*., Does a tryptophan substitution at position 56 simultaneously change *K*_*ix-Ala*_ and *Q*_*ax-Ala*_?). No correlation was observed (Supplemental Fig. 6). Furthermore, when the maximum rheostat and toggle scores were examined for each position (Fig. 8), all positions except 445 had either a high rheostat score or a high toggle score for at least one parameter. Moreover, positions 482 and 494 were shown to exert rheostatic control over one function and toggle control over a second. Thus, it appears that substitutions at individual hLPYK positions can alter multiple functional parameters.

## Discussion

Previously, we identified rheostat positions in the two allosteric sites of hLPYK and showed that allosteric coupling between their respective ligands and substrate binding was fully tunable *via* substitutions at these subsets of positions. In other words, PEP/Ala allosteric coupling was tunable *via* substitutions in the Ala binding site and PEP/Fru-1,6-BP allosteric coupling was tunable *via* substitutions in the Fru-1,6-BP binding site^3^. Full tunability was indicated by the range of functional changes that resulted from substitutions (Fig. 2) that resulted in a rheostat score near 1. The goal of this current study was to determine whether positions distant from the two allosterically-coupled binding sites could also exert rheostatic control over allosteric coupling. To that end, we used the identical variants of the previous study and evaluated their effects on the allosteric parameters of the other binding site.

The results from this study confirm that substitutions distant from the two allosterically-coupled binding sites can exert full tunability over allosteric coupling (*e.g*., full bin occupancies in Fig. 5 and rheostat scores of 1 in Fig. 6). Arguably, this study was biased towards success since the chosen “distant” positions were located in sites with known regulatory function. Nonetheless, it is striking that substitutions at these positions have as much or more rheostatic control of the distant parameters as they do their local parameters (Supplemental Fig. 5). Future substitution studies at positions distant to any known allosteric binding sites will be useful to evaluate whether this influence is restricted to allosteric binding sites or common to many regions throughout the protein. We find it possible that rheostatic control of allosteric parameters will be widespread throughout the protein.

The scores shown in Fig. 4 exhibit asymmetry in the distant effects on the allosteric parameters. Likewise, the distributions shown in Figs. 2 and 5 show that effects of substitutions in the Ala binding site were more evenly distributed over the available range than were the outcomes from substitutions in the Fru-1,6-BP binding site. A trivial explanation for the different distributions is incomplete sampling: We did not assess all possible substitutions at each position. Alternatively, the observed asymmetries may support that only a few positions (and potentially only a few side-chain types at those positions) in one area of a protein (*i.e*., a few in the Fru-1,6-BP binding site) are energetically coupled to many positions in other regions of the protein (*i.e*., the alanine binding site). This idea is consistent with the identification of “chokepoint” positions in mechanical coupling studies^21^ and observations about asymmetric dynamic coupling among protein positions involved in allosteric regulation^22^. For hLPYK, the chokepoint predictions are consistent with outcomes from a full protein alanine scan^20^. If the asymmetry persists as a feature of hLPYK allosteric regulation, the next question will be to determine whether there has been evolutionary pressure on the enzyme to use this asymmetry for a particular role in its biology, or whether the asymmetry is a fundamental and generalizable mechanism that gives rise to allosteric regulation of protein function.

Another asymmetry is obvious when comparing the distant effects on *Q*_*ax*_ values to those on *K* values. The effects on *Q*_*ax*_ values were consistently much larger. This observation agrees with the emerging view that the positions which contribute to binding can be distinct from those that contribute to allosteric regulation^5,8,23,24^.

We anticipate that understanding tunability of function *via* substitution will facilitate better ways to rationally engineer allosteric communication in new proteins and to identify potential relationships between allostery and genetic disease in personalized medicine. Indeed, this work can be summarized as a study of the tunability (*via* substitutions at rheostat positions) of tunability (allosteric regulation). As we pursue the twin goals of protein engineering and disease prediction, we must keep in mind two striking observations from these studies: (1) One position can have rheostatic control over two or more functional parameters. (2) One position can have rheostatic control of one functional parameter and toggle control over a second (Figs. 7 and 8). The need to optimize multiple functions in protein engineering has already been realized in several systems^25–29^. Based on the outcomes from this study, dual optimizations may be possible with single substitutions that are distant from ligand binding sites.

In summary, our attempt to quantitatively evaluate the toggle/rheostat/neutral character of even a limited number of positions in one protein has highlighted that allosteric coupling is highly tunable, even by positions distant from the two allosterically-coupled binding sites. Furthermore, the overlapping roles for one position in controlling multiple functions illuminates the challenges that must be resolved for protein engineering and effective predictions of functional outcomes from variants in patient genomes.

### Relevant guidelines

All use of recombinant DNA was according to the NIH Guidelines and was approved by the KUMC Institutional Biosafety Committee.

## Supplementary information

Supplementary Information

## Data Availability

All data generated or analyzed during this study are included in this published article and its Supplementary Information file.
