# Supplementary material for: Functional tunability from a distance: Rheostat positions influence allosteric coupling between two distant binding sites
Source: Sci Rep. 2019 Nov 18;9:16957. doi: 10.1038/s41598-019-53464-z (PMC6861286; doi:10.1038/s41598-019-53464-z)
Supplement: Supplementary file 1 — Supplementary Information [file 41598_2019_53464_MOESM1_ESM.pdf]

Supplemental Information

Functional tunability from a distance: Rheostat positions influence allosteric coupling between two distant binding sites

Tiffany Wu[1], Liskin Swint-Kruse[1] and Aron W. Fenton[1,*]

Supplemental Table 1: Averages from wildtype replicates		
	**average in mM (n=5)**	**Standard deviation of independent evaluations**
$K_{a\text{-}PEP}$ (mM)	0.24	0.02
$K_{ix\text{-}ala}$ (mM)	0.31	0.04
$K_{ix\text{-}FBP}$ (mM)	0.00018	0.00008
$Q_{ax\text{-}ala}$	0.073	0.009
$Q_{ax\text{-}FBP}$	14	3

Supplemental Figures 1-4. The figures below depict the raw data from Supplemental Tables 2 and 3 on log scale (left column of panels) and as histograms (right column of panels) for each parameter of each variant at each position described in this study. On plots with raw data, variants for which some activity could be detected but the plateau could not be reached are represented with the bars near threshold indicated by the dotted line. This value was also used to designate the "dead" value in histogram analyses. On the histograms, a white dot is used to indicate the bin that includes wildtype data and a black dot is used to indicate the bin that corresponds to "dead" (e.g., "no allostery") function.

Supplemental Figure 1. $K_{ix\text{-}Ala}$ individual histograms

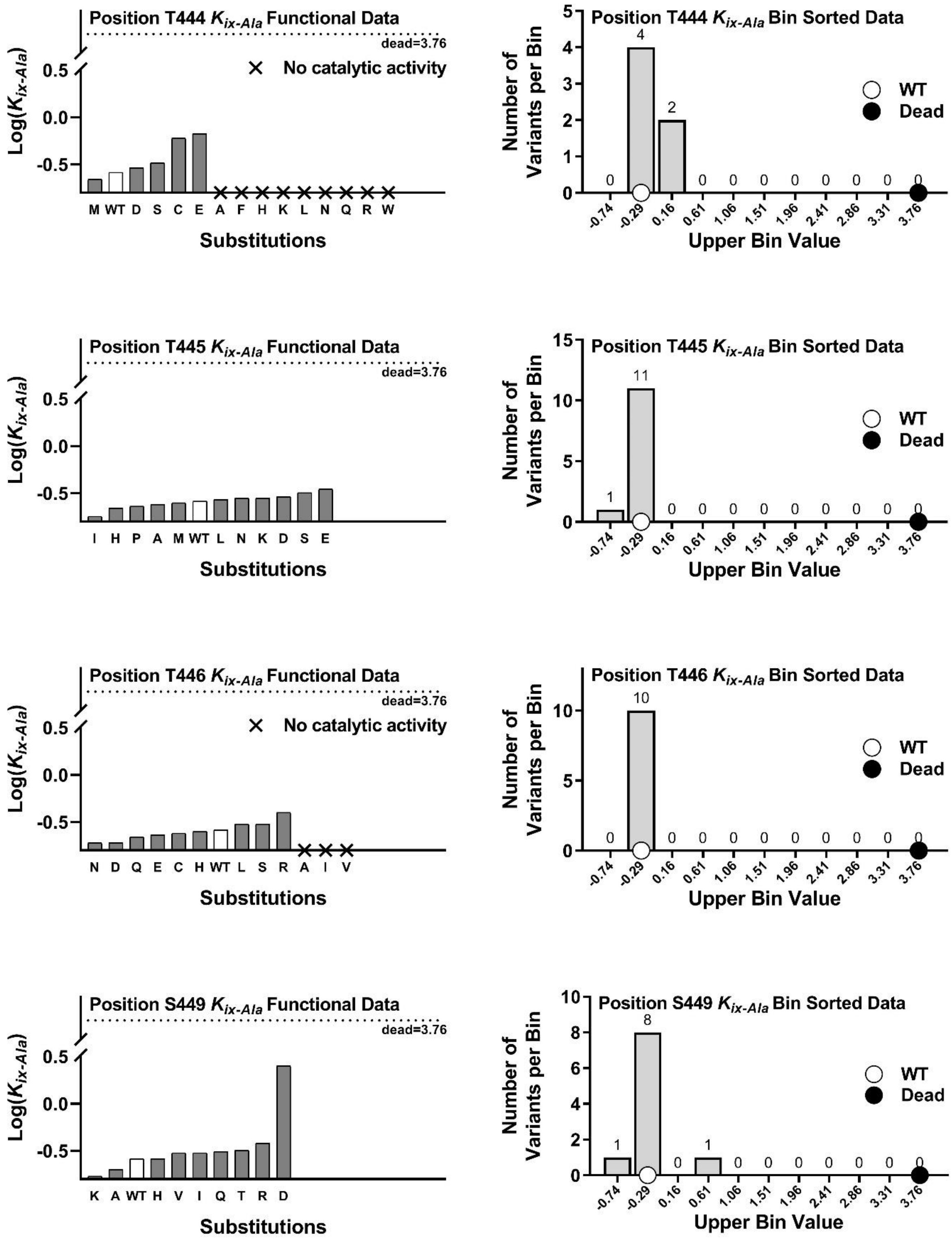

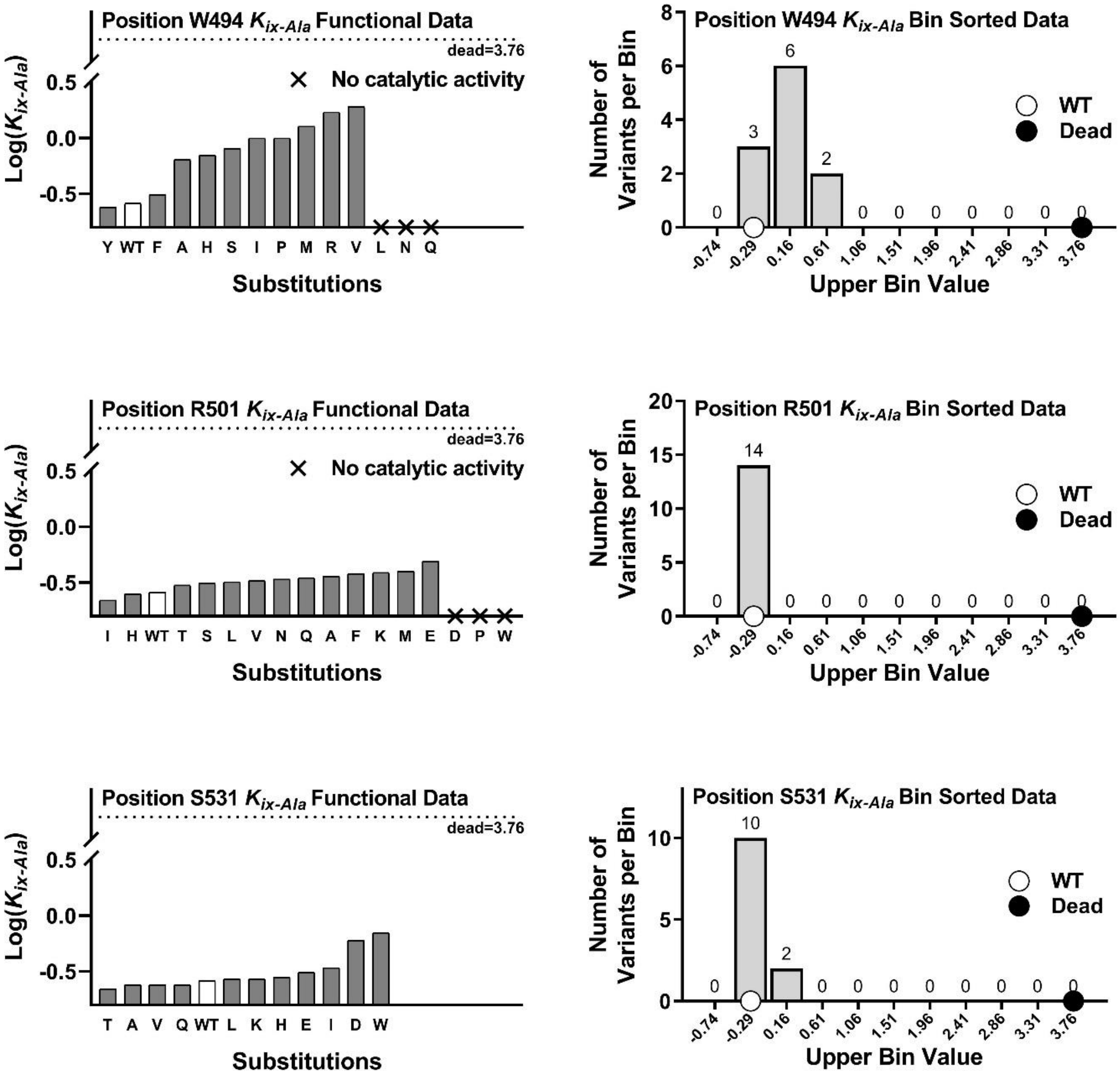

Position W494 K_{ix-Ala} Functional Data
dead=3.76
No catalytic activity
Log(K_{ix-Ala})
0.5
0.0
-0.5
Y WT F A H S I P M R V L N Q
Substitutions
Position W494 K_{ix-Ala} Bin Sorted Data
Number of Variants per Bin
8
6
4
2
0
0 3 6 2 0 0 0 0 0 0 0
WT
Dead
-0.74 -0.29 0.16 0.61 1.06 1.51 1.96 2.41 2.86 3.31 3.76
Upper Bin Value
Position R501 K_{ix-Ala} Functional Data
dead=3.76
No catalytic activity
Log(K_{ix-Ala})
0.5
0.0
-0.5
I H WT T S L V N Q A F K M E D P W
Substitutions
Position R501 K_{ix-Ala} Bin Sorted Data
Number of Variants per Bin
20
15
10
5
0
0 14 0 0 0 0 0 0 0 0 0
WT
Dead
-0.74 -0.29 0.16 0.61 1.06 1.51 1.96 2.41 2.86 3.31 3.76
Upper Bin Value
Position S531 K_{ix-Ala} Functional Data
dead=3.76
Log(K_{ix-Ala})
0.5
0.0
-0.5
T A V Q WT L K H E I D W
Substitutions
Position S531 K_{ix-Ala} Bin Sorted Data
Number of Variants per Bin
10
5
0
0 10 2 0 0 0 0 0 0 0 0
WT
Dead
-0.74 -0.29 0.16 0.61 1.06 1.51 1.96 2.41 2.86 3.31 3.76
Upper Bin Value

Supplemental Figure 2. $K_{ix\text{-}FBP}$ individual histograms

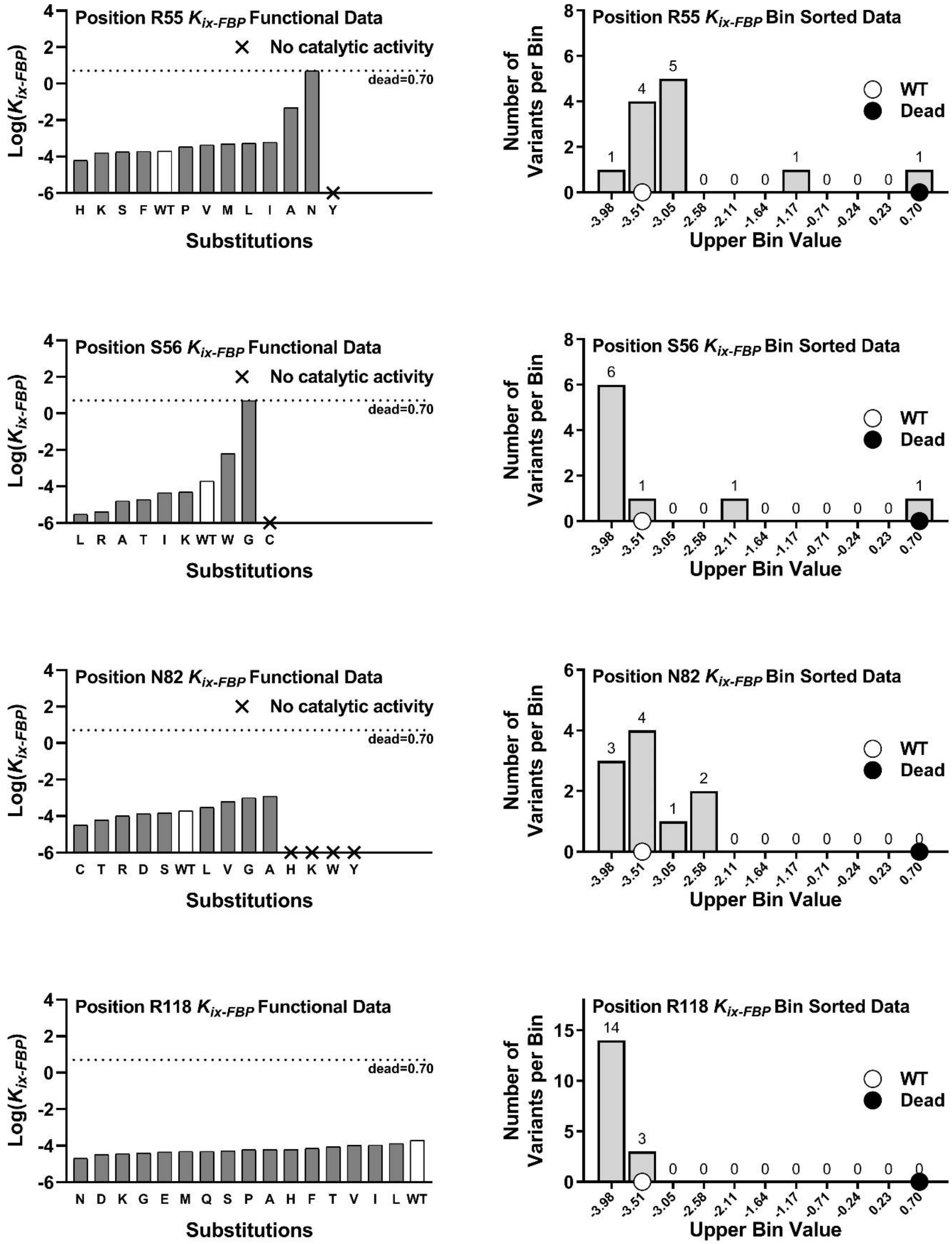

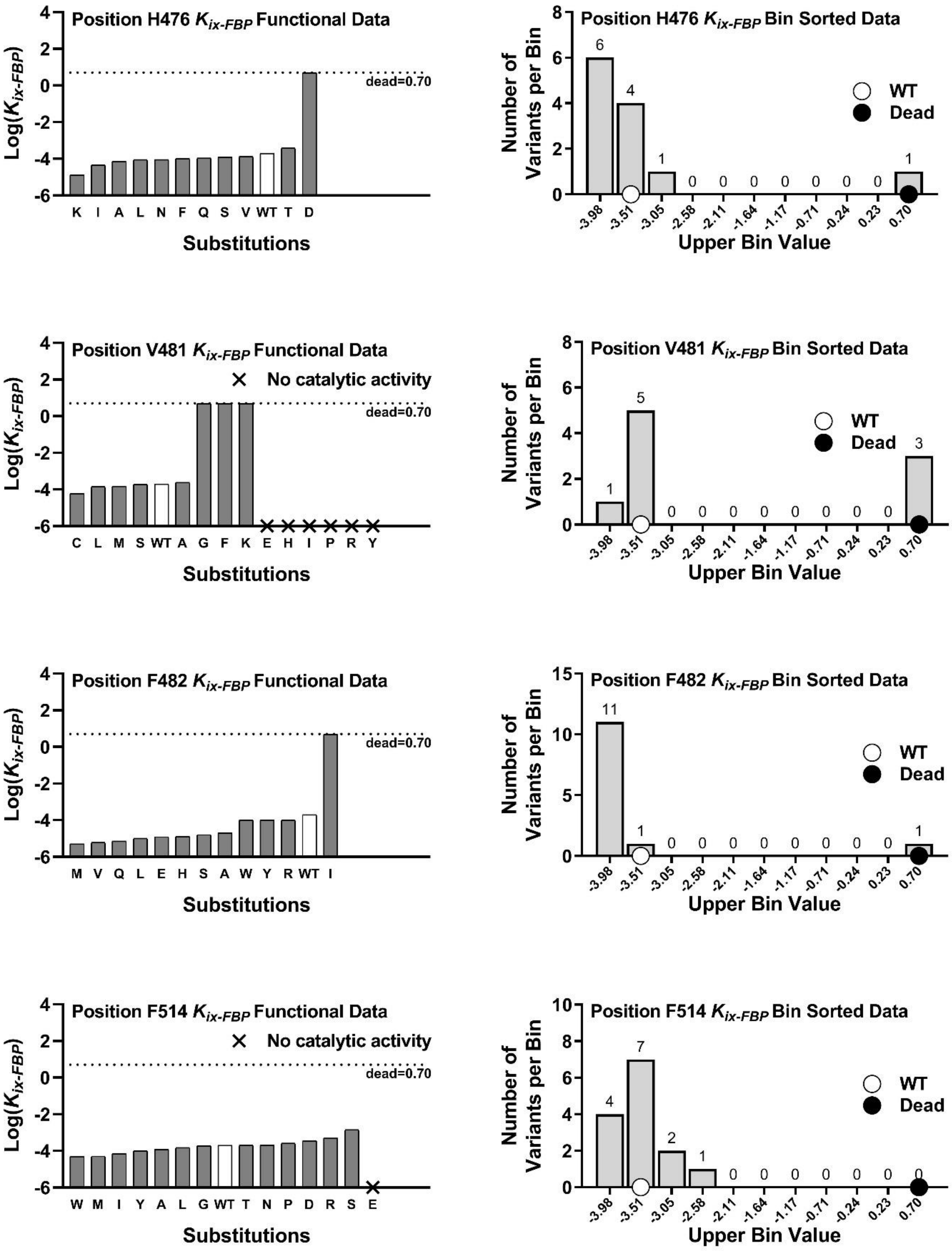

Position H476 K_{ix-FBP} Functional Data
Log(K_{ix-FBP})
dead=0.70
K I A L N F Q S V WT T D
Substitutions
Position H476 K_{ix-FBP} Bin Sorted Data
Number of Variants per Bin
6 4 1 0 0 0 0 0 0 0 1
WT
Dead
-3.98 -3.51 -3.05 -2.58 -2.11 -1.64 -1.17 -0.71 -0.24 0.23 0.70
Upper Bin Value
Position V481 K_{ix-FBP} Functional Data
× No catalytic activity
Log(K_{ix-FBP})
dead=0.70
C L M S WT A G F K E H I P R Y
Substitutions
Position V481 K_{ix-FBP} Bin Sorted Data
Number of Variants per Bin
1 5 0 0 0 0 0 0 0 0 3
WT
Dead
-3.98 -3.51 -3.05 -2.58 -2.11 -1.64 -1.17 -0.71 -0.24 0.23 0.70
Upper Bin Value
Position F482 K_{ix-FBP} Functional Data
Log(K_{ix-FBP})
dead=0.70
M V Q L E H S A W Y R WT I
Substitutions
Position F482 K_{ix-FBP} Bin Sorted Data
Number of Variants per Bin
11 1 0 0 0 0 0 0 0 0 1
WT
Dead
-3.98 -3.51 -3.05 -2.58 -2.11 -1.64 -1.17 -0.71 -0.24 0.23 0.70
Upper Bin Value
Position F514 K_{ix-FBP} Functional Data
× No catalytic activity
Log(K_{ix-FBP})
dead=0.70
W M I Y A L G WT T N P D R S E
Substitutions
Position F514 K_{ix-FBP} Bin Sorted Data
Number of Variants per Bin
4 7 2 1 0 0 0 0 0 0 0
WT
Dead
-3.98 -3.51 -3.05 -2.58 -2.11 -1.64 -1.17 -0.71 -0.24 0.23 0.70
Upper Bin Value

Supplemental Figure 3. $Q_{ax\text{-}Ala}$ individual histograms

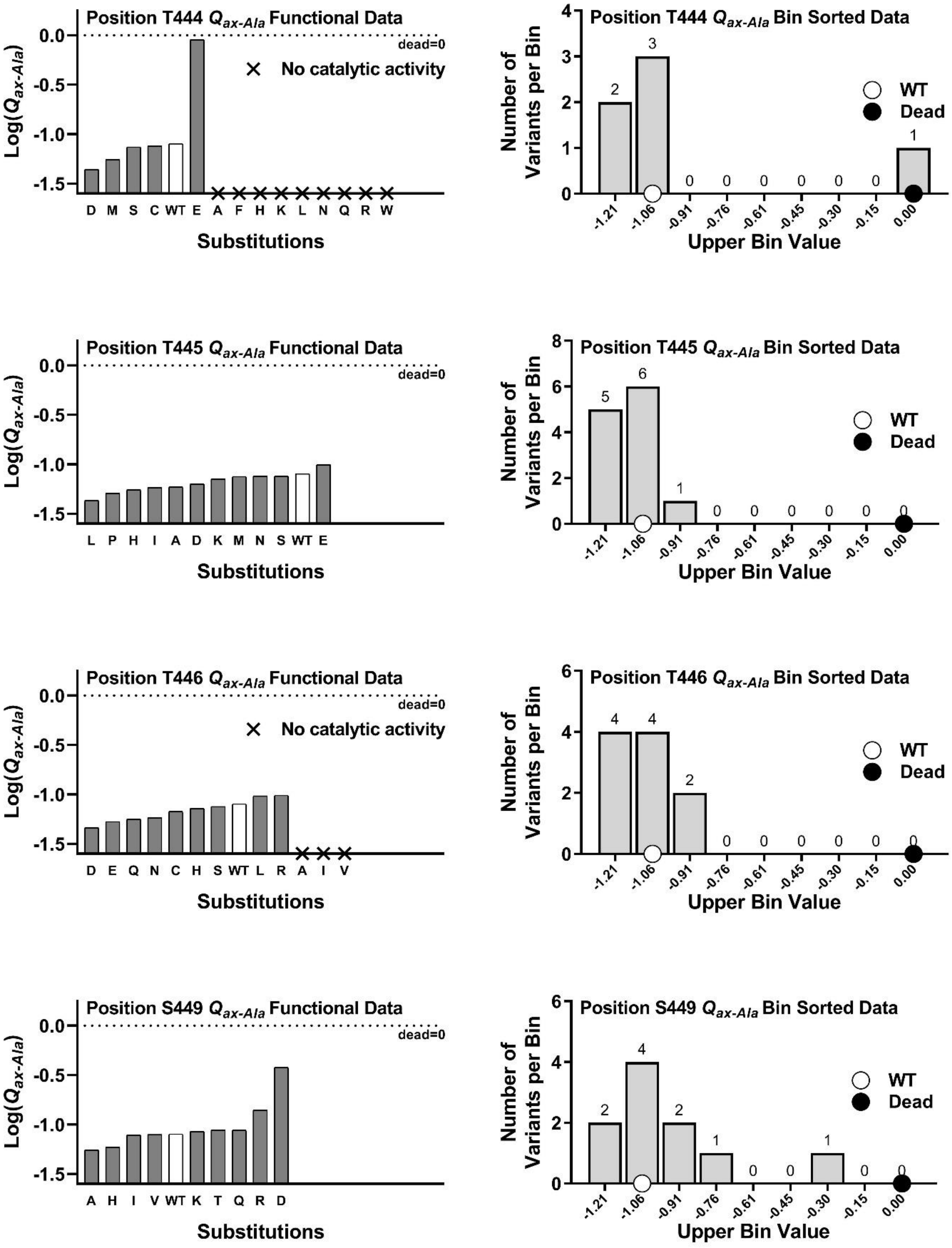

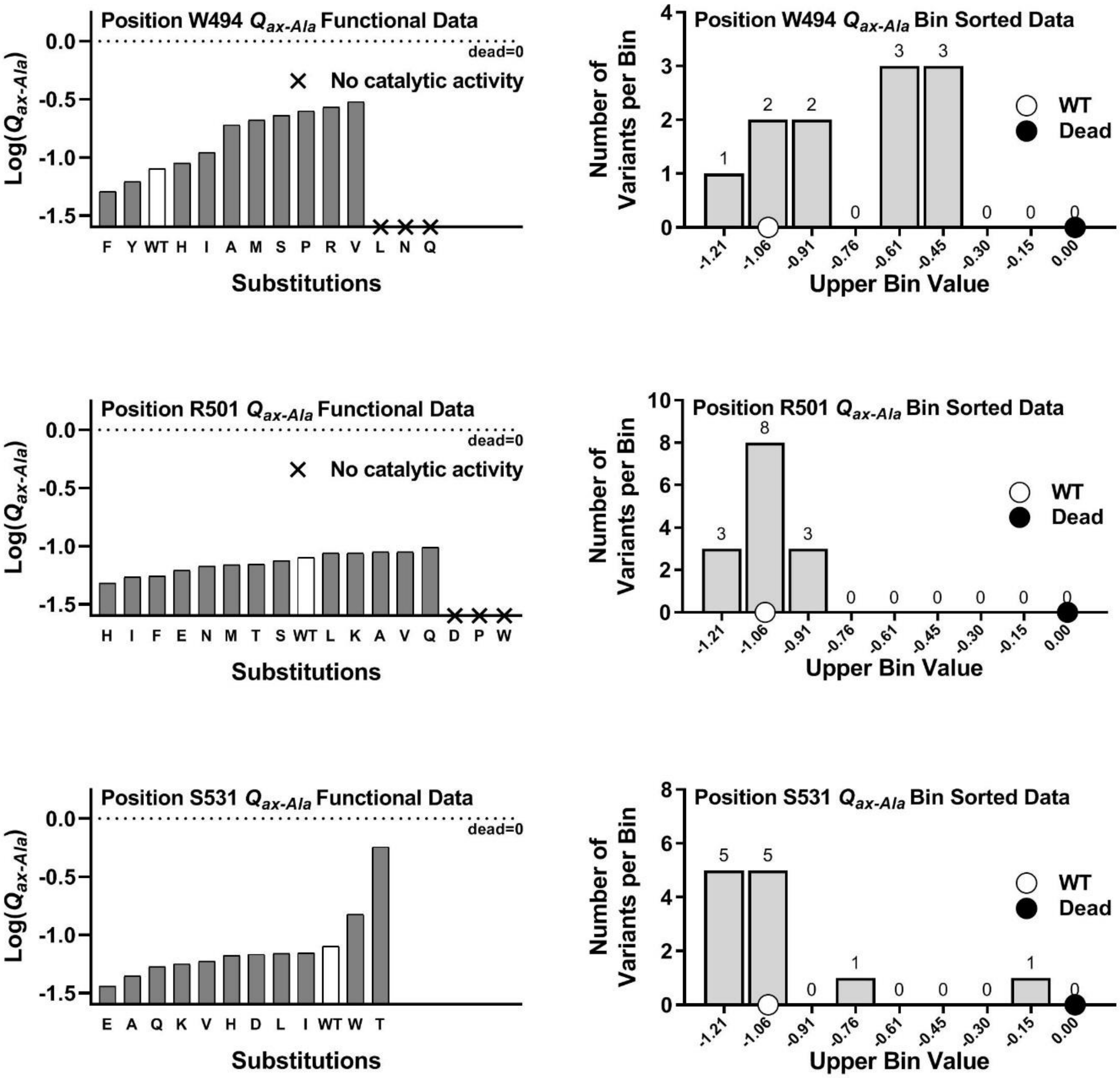

Position W494 Q_{ax-Ala} Functional Data
Log(Q_{ax-Ala})
0.0
-0.5
-1.0
-1.5
dead=0
× No catalytic activity
F Y WT H I A M S P R V L N Q
Substitutions
Position W494 Q_{ax-Ala} Bin Sorted Data
Number of Variants per Bin
1 2 2 0 3 3 0 0 0
WT
Dead
-1.21 -1.06 -0.91 -0.76 -0.61 -0.45 -0.30 -0.15 0.00
Upper Bin Value
Position R501 Q_{ax-Ala} Functional Data
Log(Q_{ax-Ala})
0.0
-0.5
-1.0
-1.5
dead=0
× No catalytic activity
H I F E N M T S WT L K A V Q D P W
Substitutions
Position R501 Q_{ax-Ala} Bin Sorted Data
Number of Variants per Bin
3 8 3 0 0 0 0 0 0
WT
Dead
-1.21 -1.06 -0.91 -0.76 -0.61 -0.45 -0.30 -0.15 0.00
Upper Bin Value
Position S531 Q_{ax-Ala} Functional Data
Log(Q_{ax-Ala})
0.0
-0.5
-1.0
-1.5
dead=0
E A Q K V H D L I WT W T
Substitutions
Position S531 Q_{ax-Ala} Bin Sorted Data
Number of Variants per Bin
5 5 0 1 0 0 0 1 0
WT
Dead
-1.21 -1.06 -0.91 -0.76 -0.61 -0.45 -0.30 -0.15 0.00
Upper Bin Value

Supplemental Figure 4. $Q_{ax\text{-}FBP}$ individual histograms

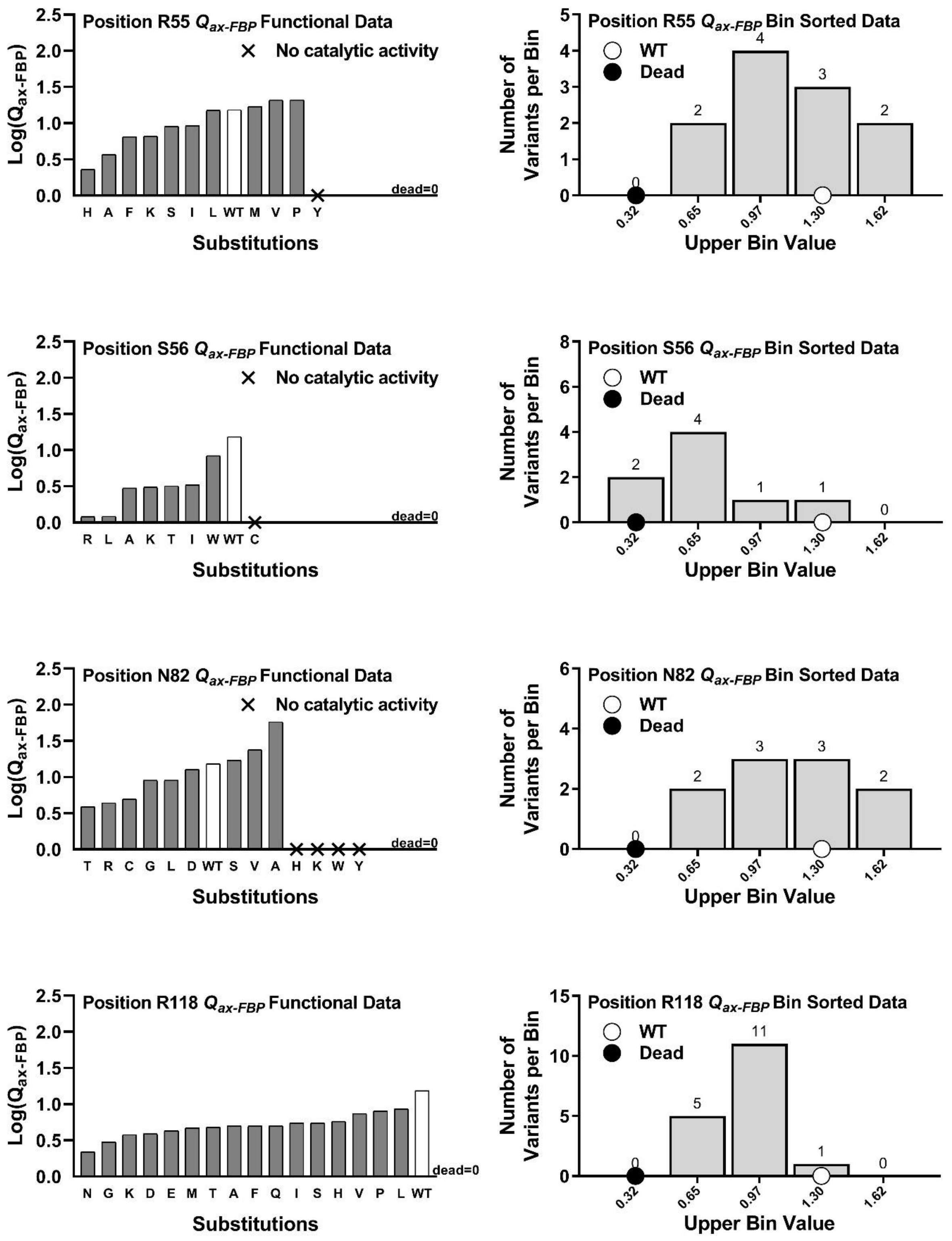

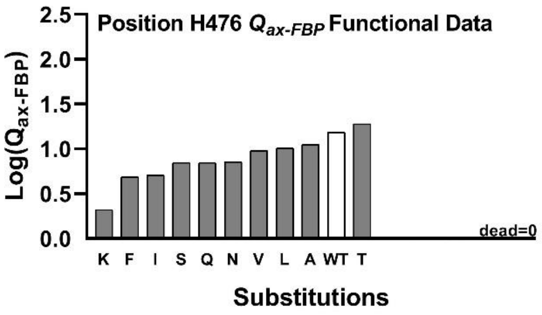
Position H476 $Q_{ax\text{-}FBP}$ Functional Data
Log($Q_{ax\text{-}FBP}$)
2.5
2.0
1.5
1.0
0.5
0.0
dead=0
K F I S Q N V L A WT T
Substitutions

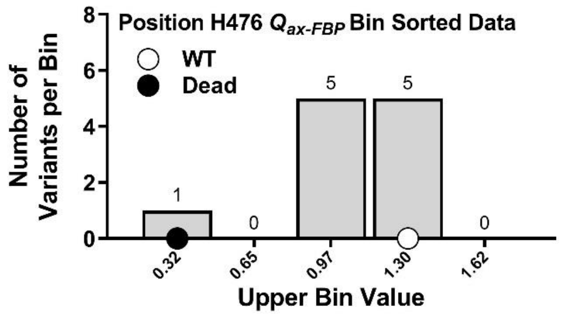
Position H476 $Q_{ax\text{-}FBP}$ Bin Sorted Data
WT
Dead
Number of Variants per Bin
8
6
4
2
0
1 0 5 5 0
0.32 0.65 0.97 1.30 1.62
Upper Bin Value

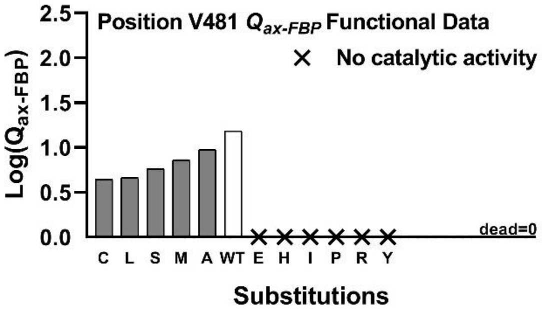
Position V481 $Q_{ax\text{-}FBP}$ Functional Data
× No catalytic activity
Log($Q_{ax\text{-}FBP}$)
2.5
2.0
1.5
1.0
0.5
0.0
dead=0
C L S M A WT E H I P R Y
Substitutions

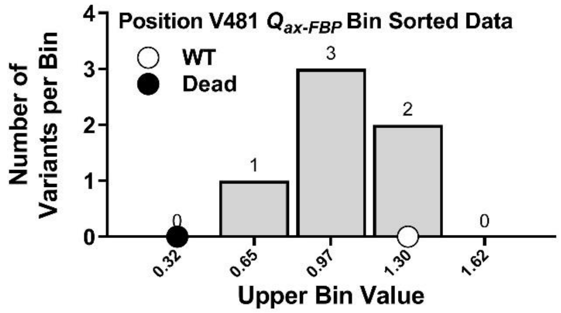
Position V481 $Q_{ax\text{-}FBP}$ Bin Sorted Data
WT
Dead
Number of Variants per Bin
4
3
2
1
0
0 1 3 2 0
0.32 0.65 0.97 1.30 1.62
Upper Bin Value

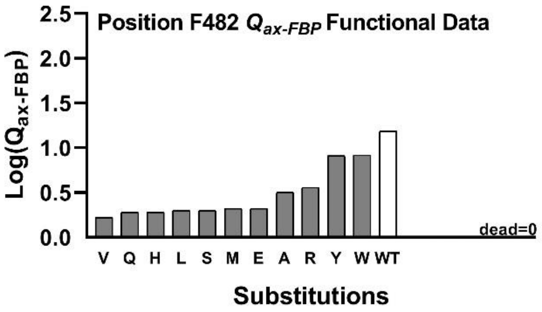
Position F482 $Q_{ax\text{-}FBP}$ Functional Data
Log($Q_{ax\text{-}FBP}$)
2.5
2.0
1.5
1.0
0.5
0.0
dead=0
V Q H L S M E A R Y W WT
Substitutions

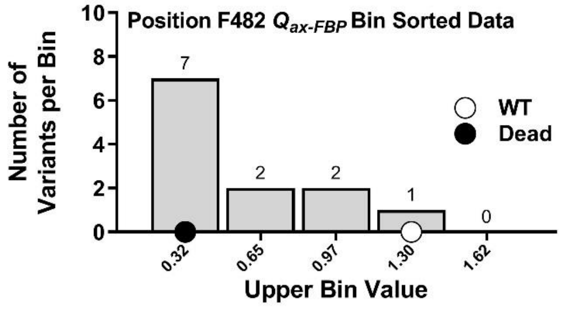
Position F482 $Q_{ax\text{-}FBP}$ Bin Sorted Data
WT
Dead
Number of Variants per Bin
10
8
6
4
2
0
7 2 2 1 0
0.32 0.65 0.97 1.30 1.62
Upper Bin Value

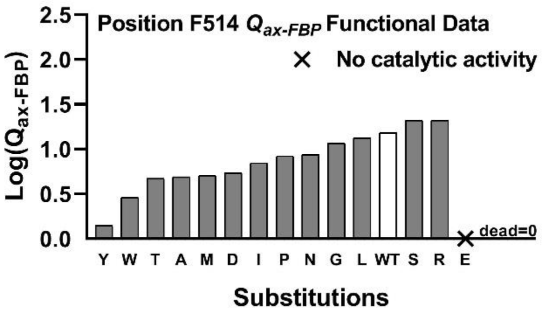
Position F514 $Q_{ax\text{-}FBP}$ Functional Data
× No catalytic activity
Log($Q_{ax\text{-}FBP}$)
2.5
2.0
1.5
1.0
0.5
0.0
dead=0
Y W T A M D I P N G L WT S R E
Substitutions

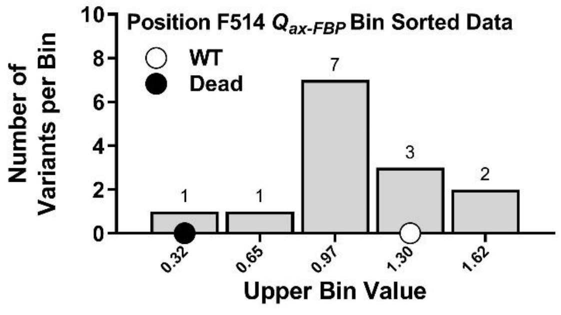
Position F514 $Q_{ax\text{-}FBP}$ Bin Sorted Data
WT
Dead
Number of Variants per Bin
10
8
6
4
2
0
1 1 7 3 2
0.32 0.65 0.97 1.30 1.62
Upper Bin Value

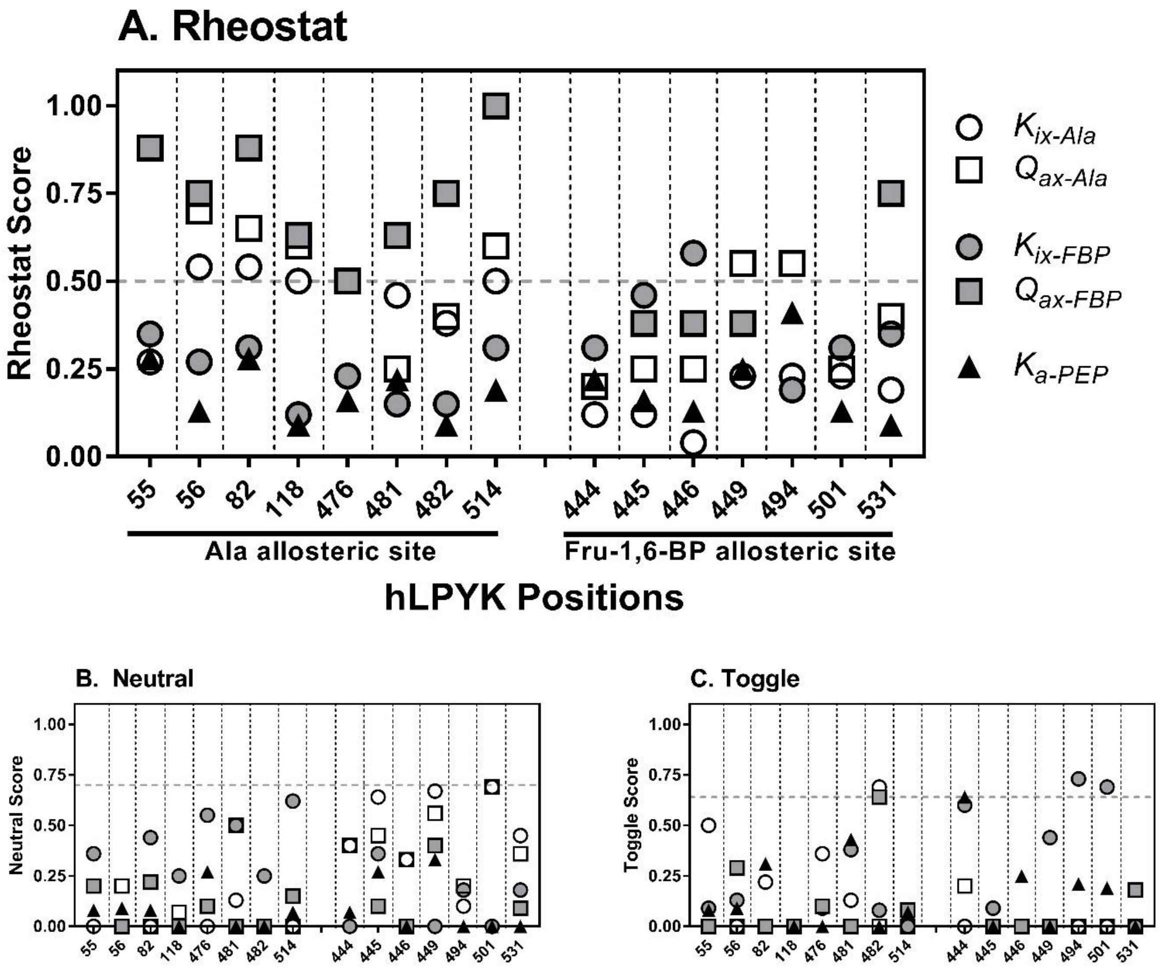

Supplemental Figure 5. All scores for local and distant evaluations were combined to assess the overall functional role of each position.

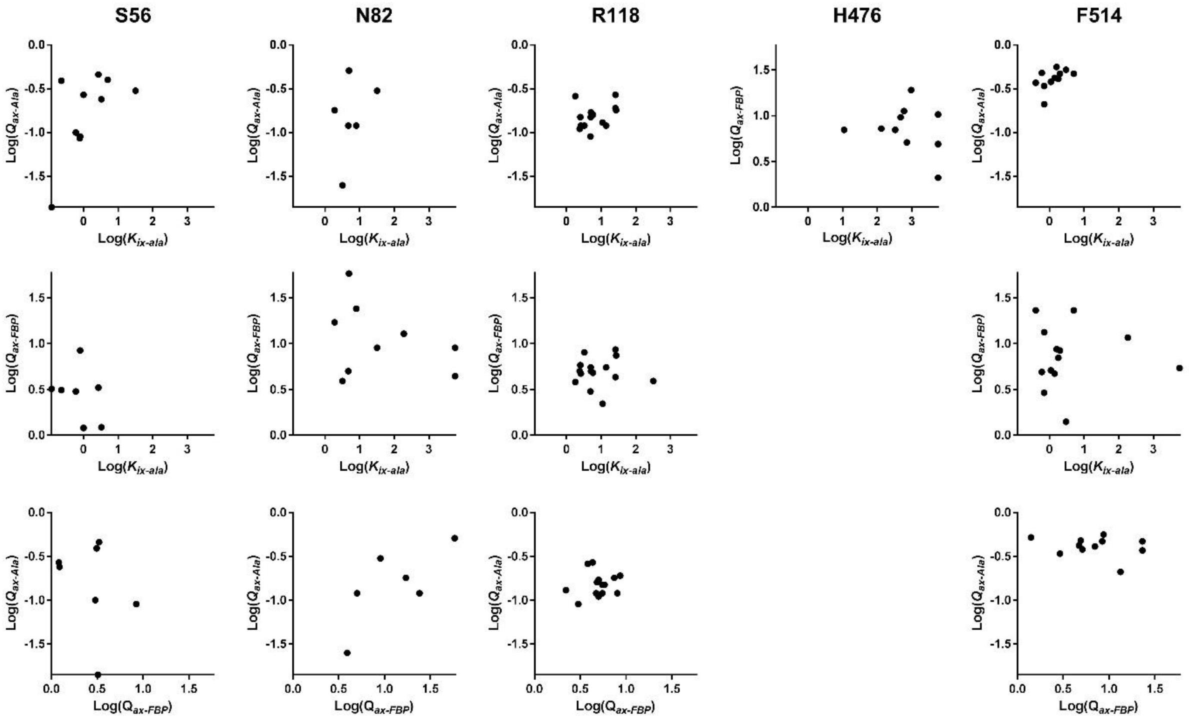

Supplemental Figure 6. Correlation between parameters. For positions with two or more rheostat scores greater than 0.5, the values of the relevant $K_{a\text{-}PEP}$, $K_{ix\text{-}Ala}$, $K_{ix\text{-}FBP}$, $Q_{ax\text{-}Ala}$, or $Q_{ax\text{-}FBP}$ were compared for each variant (individual dots). No correlation is observed among the parameters, which indicates that each substitution has independent effects on the different functional parameters.

Supplemental Table 2

Protein	$K_{a\text{-}PEP}$ [mM]	$K_{ix\text{-}Ala}$ [mM]	$Q_{ax\text{-}Ala}$
Wildtype[b]	0.23±0.01	0.26±0.01	0.080±0.001
**Thr444**			
T444A	No activity	No activity	No activity
T444L	No activity	No activity	No activity
T444M	0.38±0.01	0.22±0.02	0.055±0.003
T444F	No activity	No activity	No activity
T444W	No activity	No activity	No activity
T444S	0.25±0.01	0.33±0.03	0.074±0.003
T444C	0.70±0.06	0.6±0.1	0.076±0.007
T444N	No activity	No activity	No activity
T444Q	No activity	No activity	No activity
T444H	No activity	No activity	No activity
T444K	No activity	No activity	No activity
T444R	No activity	No activity	No activity
T444D	0.40±0.01	0.29±0.03	0.044±0.002
T444E	0.66±0.02	0.67±0.02	0.9±0.1
**Thr445**			
T445A	0.16±0.01	0.24±0.01	0.059±0.002
T445L	0.24±0.01	0.27±0.01	0.043±0.001
T445I	0.15±0.01	0.18±0.01	0.058±0.002
T445M	0.21±0.01	0.25±0.01	0.075±0.002
T445P	0.44±0.01	0.23±0.02	0.051±0.007
T445S	0.20±0.01	0.32±0.02	0.076±0.003
T445N	0.21±0.01	0.28±0.01	0.076±0.001
T445H	0.20±0.01	0.22±0.01	0.055±0.001
T445K	0.19±0.01	0.28±0.01	0.071±0.002
T445D	0.22±0.01	0.29±0.01	0.063±0.002
T445E	0.26±0.01	0.35±0.01	0.099±0.001
**Thr446**			
T446A	No activity	No activity	No activity
T446V	No activity	No activity	No activity
T446L	0.11±0.01	0.30±0.02	0.096±0.004
T446I	No activity	No activity	No activity
T446S	0.20±0.01	0.30±0.01	0.075±0.002
T446C	0.20±0.01	0.24±0.02	0.067±0.004
T446N	0.15±0.01	0.19±0.01	0.058±0.002
T446Q	0.12±0.01	0.22±0.05	0.056±0.006
T446H	0.20±0.01	0.25±0.01	0.072±0.001
T446R	0.20±0.01	0.40±0.02	0.098±0.003

T446D	0.21±0.01	0.19±0.01	0.046±0.002
T446E	0.20±0.01	0.23±0.02	0.053±0.002
**Ser449**			
S449A	0.25±0.01	0.20±0.01	0.055±0.001
S449V	0.32±0.01	0.30±0.02	0.079±0.003
S449I	0.23±0.01	0.30±0.03	0.078±0.004
S449T	0.18±0.01	0.32±0.03	0.088±0.004
S449Q	0.25±0.01	0.31±0.02	0.088±0.003
S449H	0.17±0.01	0.26±0.02	0.059±0.002
S449K	0.12±0.01	0.17±0.01	0.085±0.004
S449R	0.43±0.01	0.38±0.03	0.14±0.01
S449D	1.2±0.1	2.5±0.3	0.38±0.01
**Trp494**			
W494A	0.91±0.02	0.64±0.07	0.19±0.01
W494V	1.71±0.05	1.9±0.3	0.30±0.01
W494L	No activity	No activity	No activity
W494I	0.89±0.05	1.0±0.2	0.11±0.01
W494M	1.29±0.01	1.27±0.05	0.21±0.01
W494P	1.42±0.03	1.0±0.1	0.25±0.01
W494F	0.10±0.01	0.31±0.03	0.051±0.002
W494S	1.27±0.07	0.8±0.2	0.23±0.01
W494Y	0.14±0.01	0.24±0.01	0.062±0.002
W494N	No activity	No activity	No activity
W494Q	No activity	No activity	No activity
W494H	0.096±0.007	0.7±0.1	0.089±0.007
W494R	1.60±0.05	1.7±0.2	0.27±0.01
**Arg501**			
R501A	0.14±0.01	0.36±0.01	0.089±0.001
R501V	0.13±0.01	0.33±0.04	0.089±0.004
R501L	0.11±0.03	0.32±0.02	0.087±0.003
R501I	0.092±0.002	0.22±0.01	0.054±0.001
R501M	0.15±0.01	0.40±0.02	0.069±0.002
R501F	0.051±0.002	0.38±0.03	0.055±0.003
R501W	No activity	No activity	No activity
R501S	0.091±0.003	0.31±0.03	0.075±0.002
R501T	0.063±0.002	0.30±0.02	0.070±0.003
R501N	0.065±0.006	0.34±0.07	0.067±0.008
R501Q	0.17±0.01	0.35±0.01	0.098±0.002
R501H	0.051±0.002	0.25±0.03	0.048±0.002
R501K	0.20±0.01	0.39±0.01	0.087±0.002
R501D	No activity	No activity	No activity
R501E	0.12±0.01	0.49±0.03	0.062±0.002
**Ser531**			
S531A	0.096±0.003	0.24±0.01	0.044±0.002
S531V	0.11±0.01	0.24±0.03	0.059±0.004

S531L	0.13±0.01	0.27±0.02	0.069±0.002
S531I	0.11±0.01	0.34±0.03	0.070±0.003
S531W	0.32±0.03	0.70±0.13	0.15±0.01
S531T	0.17±0.01	0.22±0.01	0.57±0.002
S531Q	0.12±0.01	0.24±0.01	0.053±0.001
S531H	0.14±0.01	0.28±0.01	0.066±0.002
S531K	0.12±0.01	0.27±0.01	0.056±0.002
S531D	0.045±0.002	0.60±0.05	0.068±0.003
S531E	0.037±0.002	0.31±0.02	0.036±0.002

Supplemental Table 3

Protein	$K_{a\text{-}PEP}$ [mM]	$K_{ix\text{-}FBP}$ [mM]	$Q_{ax\text{-}FBP}$
Wildtype	0.250±0.003	0.000197±0.000001	15.3±0.3
**Arg55**			
R55A[b]	0.056±0.001	0.048±0.004	3.70±0.09
R55V	0.52±0.01	0.00043±0.00007	24±2
R55L	0.43±0.01	0.00055±0.000005	15±1
R55I	0.42±0.02	0.0006±0.0002	9.3±0.9
R55M	0.31±0.02	0.0005±0.0001	17±1
R55P	0.60±0.02	0.00035±0.00004	26±1
R55F	0.15±0.01	0.00019±0.00002	6.5±0.2
R55S	0.21±0.01	0.00018±0.00004	9.0±0.6
R55N	0.036±0.003	No activation	No activation
R55Y	No activity	No activity	No activity
R55H	0.062±0.001	0.00006±0.00001	2.3±0.1
R55K	0.14±0.01	0.00016±0.00007	6.6±0.9
**Ser56**			
S56G	0.026±0.001	No activation	No activation
S56A	0.079±0.005	0.000016±0.000005	3.0±0.2
S56L	0.026±0.001	0.000003±0.000004	1.22±0.06
S56I	0.093±0.003	0.000043±0.000008	3.3±0.1
S56W	0.29±0.01	0.006±0.001	8.4±0.9
S56T	0.069±0.002	0.000019±0.000003	3.2±0.1
S56C	No activity	No activity	No activity
S56K	0.082±0.004	0.00005±0.00002	3.1±0.2
S56R	0.022±0.001	0.000004±0.000005	1.20±0.07
**Asn82**			
N82G	0.26±0.01	0.0010±0.0004	9±2
N82A	0.99±0.01	0.0012±0.0001	58±2
N82V	0.53±0.02	0.0006±0.0001	24±3
N82L	0.26±0.01	0.0003±0.0001	9±1
N82W	No activity	No activity	No activity
N82S	0.35±0.01	0.00015±0.00003	17±1
N82T	0.082±0.007	0.00006±0.00002	3.9±0.4
N82C	0.14±0.01	0.000032±0.000005	5.0±0.2
N82Y	No activity	No activity	No activity
N82H	No activity	No activity	No activity
N82K	No activity	No activity	No activity
N82R	0.12±0.01	0.00010±0.00002	4.4±0.2
N82D	0.42±0.01	0.00013±0.00002	12.8±0.5

**Arg118**			
R118G	0.080±0.003	0.00004±0.00001	3.0±0.2
R118A	0.15±0.01	0.000062±0.000007	5.0±0.3
R118V	0.16±0.01	0.00010±0.00002	7.4±0.4
R118L	0.17±0.01	0.00013±0.00004	8.6±0.6
R118I	0.13±0.01	0.00011±0.00002	5.5±0.2
R118M	0.12±0.01	0.00005±0.00001	4.7±0.2
R118P	0.16±0.01	0.00006±0.00001	8.0±0.4
R118F	0.11±0.01	0.00007±0.00001	5.0±0.4
R118S	0.14±0.01	0.000052±0.000004	5.5±0.2
R118T	0.12±0.01	0.00009±0.00001	4.8±0.3
R118N	0.066±0.006	0.00002±0.00001	2.2±0.2
R118Q	0.13±0.01	0.00005±0.00001	5.0±0.3
R118H	0.14±0.01	0.000062±0.000008	5.8±0.2
R118K	0.099±0.004	0.000036±0.000008	3.8±0.2
R118D	0.11±0.01	0.000033±0.000004	3.9±0.1
R118E	0.12±0.01	0.000045±0.000009	4.3±0.2
**His476**			
H476A	0.27±0.01	0.000069±0.000007	11.2±0.03
H476V	0.18±0.01	0.00013±0.00002	9.6±0.9
H476L	0.22±0.01	0.00009±0.00001	10.3±0.4
H476I	0.11±0.01	0.000046±0.000006	5.1±0.2
H476F	0.12±0.01	0.00010±0.00002	4.9±0.2
H476S	0.23±0.01	0.00012±0.00003	7±1
H476T	0.44±0.01	0.00038±0.00003	19±1
H476N	0.20±0.01	0.00009±0.00002	7.2±0.4
H476Q	0.16±0.01	0.00011±0.00002	7.0±0.04
H476K	0.056±0.001	0.000013±0.000005	2.1±0.1
H476D	0.027±0.002	No activation	No activation
**Val481**			
V481G	0.024±0.001	No activation	No activation
V481A	0.21±0.01	0.00024±0.00003	9.5±0.7
V481L	0.12±0.01	0.00014±0.00008	4.6±0.7
V481I	No activity	No activity	No activity
V481M	0.24±0.02	0.00015±0.00005	7.2±0.8
V481P	No activity	No activity	No activity
V481F	0.76±0.028	No activation	No activation
V481S	0.13±0.01	0.00019±0.00006	5.8±.07
V481C	0.092±0.004	0.00006±0.00001	4.4±0.2
V481Y	No activity	No activity	No activity
V481H	No activity	No activity	No activity
V481K	0.16±0.01	No activation	No activation
V481R	No activity	No activity	No activity
V481E	No activity	No activity	No activity
**Phe482**			
F482A	0.069±0.003	0.000020±0.000005	3.2±0.2

F482V	0.033±0.001	0.000006±0.000004	1.66±0.08
F482L	0.045±0.002	0.000010±0.000004	2.0±0.1
F482I	0.032±0.002	No activation	No activation
F482M	0.038±0.002	0.000005±0.000002	2.1±0.1
F482W	0.21±0.01	0.00010±0.00001	8.3±0.4
F482S	0.041±0.001	0.000016±0.000005	2.0±0.1
F482Q	0.039±0.002	0.000007±0.000003	1.9±0.1
F482Y	0.20±0.01	0.00010±0.00002	8.1±0.5
F482H	0.048±0.003	0.000013±0.000005	1.9±0.1
F482R	0.076±0.0004	0.00010±0.00003	3.6±0.3
F482E	0.044±0.002	0.000012±0.000005	2.1±0.1
**Pro483**			
P483G	0.13±0.01	0.000045±0.000005	4.9±0.2
P483A	No activity	No activity	No activity
P483V	No activity	No activity	No activity
P483L	No activity	No activity	No activity
P483I	No activity	No activity	No activity
P483W	No activity	No activity	No activity
P483T	No activity	No activity	No activity
P483C	No activity	No activity	No activity
P483K	No activity	No activity	No activity
P483D	No activity	No activity	No activity
P483E	No activity	No activity	No activity
**Phe514**			
F514G	0.27±0.01	0.00019±0.00002	11.6±0.5
F514A	0.16±0.01	0.00012±0.00004	4.9±0.5
F514L	0.28±0.01	0.00015±0.00001	13.3±0.4
F514I	0.17±0.01	0.00007±0.00001	7.0±0.3
F514M	0.13±0.01	0.000051±0.000006	5.1±0.2
F514P	0.14±0.01	0.00026±0.00006	8.4±0.7
F514W	0.078±0.005	0.000049±0.000008	2.9±0.3
F514S	0.36±0.02	0.0014±0.0004	23±3
F514T	0.14±0.01	0.00020±0.00005	4.7±0.5
F514N	0.18±0.01	0.00020±0.00003	8.7±0.4
F514Y	0.024±0.01	0.0001±0.0001	1.4±0.1
F514R	0.40±0.03	0.0005±0.0001	23±3
F514D	0.094±0.002	0.00035±0.00008	5.4±0.4
F514E	No activity	No activity	No activity

Supplemental Table 4

	$K_{a\text{-}PEP}$			$K_{ix\text{-}Ala}$			$K_{ix\text{-}FBP}$			$Q_{ax\text{-}Ala}$			$Q_{ax\text{-}FBP}$		
	Neutral	Rheostat	Toggle	Neutral	Rheostat	Toggle	Neutral	Rheostat	Toggle	Neutral	Rheostat	Toggle	Neutral	Rheostat	Toggle
**Positional scores**															
55	0.08	0.28	0.08	0	0.27	0.5	0.36	0.35	0.09				0.2	0.88	0
56	0.09	0.13	0.09	0	0.54	0	0	0.27	0.13	0.2	0.7	0	0	0.75	0.29
82	0.08	0.28	0.31	0	0.54	0.22	0.44	0.31	0	0	0.65	0	0.22	0.88	0
118	0	0.09	0	0	0.5	0	0.25	0.12	0	0.07	0.6	0	0	0.63	0
476	0.27	0.16	0	0	0.5	0.36	0.55	0.23	0.09				0.1	0.5	0.1
481	0	0.22	0.43	0.13	0.46	0.13	0.5	0.15	0.38	0.5	0.25	0	0	0.63	0
482	0	0.09	0	0	0.38	0.69	0.25	0.15	0.08	0	0.4	0	0	0.75	0.64
514	0.07	0.19	0.07	0	0.5	0.08	0.62	0.31	0	0	0.6	0	0.15	1	0.08
444	0.07	0.22	0.64	0.4	0.12	0	0	0.31	0.6	0.4	0.2	0.2			
445	0.27	0.16	0	0.64	0.12	0	0.36	0.46	0.09	0.45	0.25	0	0.1	0.38	0
446	0	0.13	0.25	0.33	0.04	0	0	0.58	0	0.33	0.25	0	0	0.38	0
449	0.33	0.25	0	0.67	0.23	0	0	0.38	0.44	0.56	0.55	0	0.4	0.38	0
494	0	0.41	0.21	0.1	0.23	0	0.18	0.19	0.73	0.2	0.55	0			
501	0	0.13	0.19	0.69	0.23	0	0	0.31	0.69	0.69	0.25	0			
531	0	0.09	0	0.45	0.19	0	0.18	0.35	0	0.36	0.4	0	0.09	0.75	0.18
**Summed scores**															
All	0.07	0.56	0.2	0.21	1	0.12	0.26	0.92	0.2	0.29	1	0.01	0.09	1	0.11
Local				0.01	1	0.25	0.12	0.81	0.36	0.11	0.95	0	0.1	1	0.05
Distant				0.49	0.31	0	0.37	0.58	0.08	0.44	1	0.01	0.09	1	0.13
